# CsrA impacts survival of *Yersinia enterocolitica* by affecting a myriad of physiological activities

**DOI:** 10.1186/s12866-015-0343-6

**Published:** 2015-02-14

**Authors:** Karen LeGrand, Shane Petersen, Yan Zheng, Kang K Liu, Gulustan Ozturk, Jing-Yu Chen, Glenn M Young

**Affiliations:** Microbiology Graduate Group, University of California, Davis, CA USA; Department of Food Science and Technology, University of California, Davis, Davis, CA USA; College of Food Science, Shenyang Agricultural University, Shenyang, PR China; College of Food Science and Nutritional Engineering, China Agricultural University, Beijing, China

**Keywords:** *Yersinia*, CsrA, Csr system, Motility, Salt sensitivity, Antibiotic sensitivity, Temperature sensitivity, Psychrotroph, Mutant selection

## Abstract

**Background:**

A previous study identified a *Yersinia enterocolitica* transposon mutant, GY448, that was unable to export the flagellar type three secretion system (T3SS)-dependent phospholipase, YplA. This strain was also deficient for motility and unable to form colonies on Lauria-Bertani agar medium. Preliminary analysis suggested it carried a mutation in *csrA*. CsrA in *Escherichia coli* is an RNA-binding protein that is involved in specific post-transcriptional regulation of a myriad of physiological activities. This study investigated how CsrA affects expression of the flagellar regulatory cascade that controls YplA export and motility. It also explored the effect of *csrA* mutation on *Y. enterocolitica* in response to conditions that cue physiological changes important for growth in environments found both in nature and the laboratory.

**Results:**

The precise location of the transposon insertion in GMY448 was mapped within *csrA*. Genetic complementation restored disruptions in motility and the YplA export phenotype (Yex), which confirmed this mutation disrupted CsrA function. Mutation of *csrA* affected expression of *yplA* and flagellar genes involved in flagellar T3SS dependent export and motility by altering expression of the master regulators *flhDC*. Mutation of *csrA* also resulted in increased sensitivity of *Y. enterocolitica* to various osmolytes, temperatures and antibiotics.

**Conclusions:**

The results of this study reveal unique aspects of how CsrA functions in *Y. enterocolitica* to control its physiology. This provides perspective on how the Csr system is susceptible to adaptation to particular environments and bacterial lifestyles.

## Background

*Yersinia enterocolitica* produces a phospholipase, YplA, that is secreted by the flagellar type 3 secretion system (T3SS) under standard laboratory conditions and can also be exported by the Ysa and Ysc T3SS under different conditions [1,2]. In a previous study, our laboratory developed a transposon mutant library that identified 77 mutants that exhibited a deficiency for YplA export phenotype (Yex) under standard conditions [3]. Three of the mutants carried an insertion of the transposon within the *yplA* locus. Among the remaining Yex^**−**^ strains, 74 of these mutants additionally exhibited defects for motility. Subsequent analysis confirmed that the insertion mutation harbored by 71 of these Yex^**−**^ strains mapped to genes encoding components of the flagellar T3SS (unpublished data). This result corroborated results from previous studies that established YplA export depends on this T3SS [2,4]. Two of the remaining Yex^**−**^ mutants were affected for production and sensing of the ubiquitous signaling molecule **c**yclic **AMP** (cAMP) and the **c**AMP **r**eceptor **p**rotein (CRP), which are necessary for normal expression of the flagellar, Ysa and Ysc T3SS [3]. These strains carried mutations mapping to *cya* and *crp*, respectively. The single remaining motility deficient mutant, GY448, was noted to have another striking phenotype; it was not able to grow on Lauria-Bertani (LB) agar medium, but could grow on tryptone yeast extract (TYE) agar medium. Preliminary analysis had suggested GY448 carried a transposon insertion located within a gene homologous to *Escherichia coli***c**arbon **s**torage **r**egulator **A** (*csrA*).

The *csrA* gene, and its ortholog *rsmA*, has been characterized in *E. coli* and a wide variety of other bacterial species as one that encodes an RNA-binding protein (reviewed in [5]). CsrA is involved in post-transcriptional regulation of many specific genes and consequently coordinates a myriad of physiological activities including metabolism, adaptation to changing environmental conditions and the temporal expression of colonization and virulence factors. Mechanistically, CsrA binds to target mRNAs and, depending on the context of the binding site, is capable of either activating or repressing translation [6]. CsrA function is modulated by additional components of the Csr system. Two highly structured small non-coding regulatory RNA molecules (ncRNA), CsrB and CsrC, are ncRNAs that titrate the amount of CsrA available within the cell by binding to CsrA and sequestering it from target mRNAs [6-8]. Stability of CsrB and CsrC is controlled by CsrD, adding an additional layer of modulation that ultimately affects CsrA availability [9].

## Results and discussion

A) *Y. enterocolitica* strain GY448 phenotypes can be restored by complementation of *csrA* on a low-copy plasmid.

**Figure 1:**
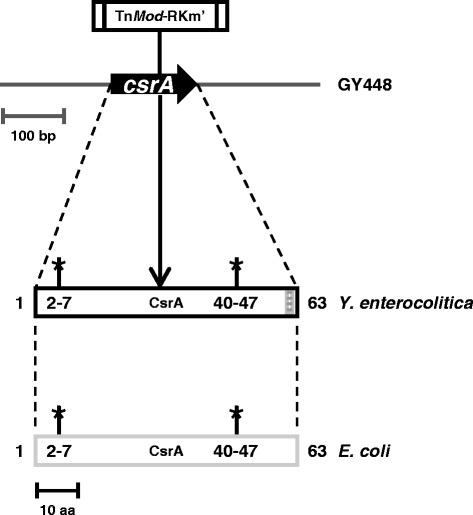
**Schematic diagram of** ***csrA*** **region in chromosome of GY448 and CsrA in** ***Y. enterocolitica*** **and** ***E. coli*** **.** The location and orientation of the *orf* is indicated by the thick black arrow. The insertion location of the transposon with the kanamycin resistance cassette (Km) is shown above. The downward arrow from this location indicates the site of the mutation within codon 29 of the protein encoded by the *Y. enterocolitica orf*, which is represented by the black rectangle. The grey rectangle represents CsrA from *E. coli*. The dashed lines between these three components indicate alignment between the *Y. enterocolitica orf*, the *Y. enterocolitica* protein and the *E. coli* protein. The stars represent regions essential for dimerization. The numbers represent amino acid position. The small grey shaded region represents non-homology of the *Y. enterocolitica* protein with the *E. coli* protein. The location of the transposon insertion results in a C-terminal truncation that excludes one of the critical regions essential for dimerization.

In order to understand the nature of the defect that affected YplA export, motility and growth of GY448 on LB media, the mutation was further characterized. The site of the transposon insertion within the *Y. enterocolitica* genome was precisely mapped. Determination of the DNA sequence of the transposon/chromosome junction revealed the location to be at codon 29 of a predicted *orf* (Figure 1). The 61 amino acid protein encoded by this *orf* is 95% identical to CsrA from *E. coli*, differing only at amino acids 58–60. This *orf* is also, 94% identical *Salmonella enterica* subsp. *enterica*, serovar Typhimurium and exhibits a high degree of amino acid similarity to various other bacteria. In *E. coli*, CsrA functions as a homodimer in which two critical regions, amino acids 2–7 and 40–47, interact in an antiparallel manner to form a functional domain [10-12]. Thus the transposon insertion was predicted to result in a null mutation since the C-terminal truncation excluded the critical dimerization domain containing residues 40–47.

**Figure 2:**
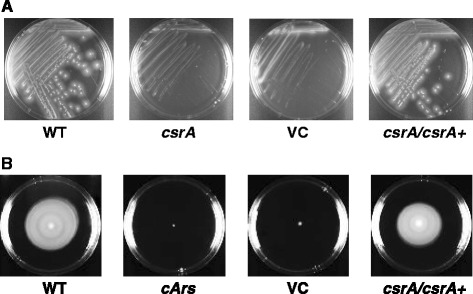
**Complementation of GY448 with** ***csrA*** **restores the Yex phenotype and motility.** GY123 (WT), GY448 (*csrA*), GY6536 (VC) and GY6535 (*csrA/csrA+*) strains were examined. **A)** The Yex phenotype was examined by determining phospholipase activity using a modified radial-diffusion assay. Individual colonies were streaked for isolation onto PLA indicator medium. Subsequently, phospholipase activity was detected as a bright white zone of precipitation emanating from isolated colonies. Representative images from three independent experiments. **B)** Phenotypic assays for motility were initiated by spotting a small portion of a colony at the center of plates containing TYE medium with 0.3% agar. Subsequently, motility was scored positive if the strains exhibited growth and migration emanating from the point of inoculation. Representative images from three independent experiments.

The prediction that CsrA in GY448 is non-functional was supported by results from genetic complementation analysis. A fragment of DNA with *csrA* was cloned into the low copy plasmid pTM100 to produce pGY1298. The plasmid was introduced into GY448, resulting in strain GY6535 (*csrA/csrA+*). As a negative control, the vector, pTM100, was also introduced into GY448, resulting in strain GY6536 (VC). These strains were examined for the ability to export YplA and for motility. The presence of pGY1298, but not pTM100 restored the Yex^**+**^ phenotype (Figure 2A) and motility (Figure 2B). These results demonstrate that the mutation carried by GY448 disrupted CsrA function.

B) *Y. enterocolitica* CsrA activates expression of genes encoding the master motility regulators FlhDC.

**Figure 3:**
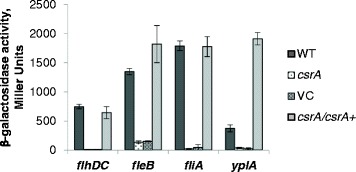
**Effect of CsrA on expression of genes within the regulatory hierarchy controlling YplA export and motility.** LacZ production from the vector pFUSE was driven by the upstream region, including the Shine-Dalgarno sequence, of *flhDC*, *fliA*, *fleB* or *yplA* in strains GY123 (WT), GY448 (*csrA*), GY6536 (VC) and GY6535 (*csrA/csrA+*). Bacterial cells were harvested and assayed for β-galactosidase activity. Results are averages ± standard deviation of at least three independent experiments performed in triplicate.

The Yex^**−**^ phenotype of the *csrA* mutant of *Y. enterocolitica* may be the result of altered *yplA* expression. Therefore, to determine whether CsrA affected *yplA* expression, a *lac* reporter system was used in which *lacZ* was driven by the promoter region of *yplA*. Gene expression in wild-type (WT) and the *csrA* mutant (*csrA*) was quantified by measuring β-galactosidase activity (Figure 3). Expression of *yplA* was significantly reduced in the *csrA* mutant relative to wild-type, indicating CsrA indeed affects *yplA* expression. The gene encoding *yplA* is one of a collection of genes within the hierarchical regulatory cascade of the flagellar T3SS of *Y. enterocolitica* defined as Class III genes [2,13]. Other Class III genes encode proteins essential for maturation of the flagellum, including the filament proteins FleA, FleB and FleC. To determine whether CsrA affected other genes within this class of flagellar genes, expression of *fleB* was also examined (Figure 3). The *csrA* mutant expressed significantly less *fleB* than wild-type, indicating the effect of CsrA on Class III genes was not limited to *yplA*. Considering that CsrA affected expression of two different Class III genes, we reasoned that CsrA may act at a higher level within the regulatory cascade. Class III genes are regulated by a sigma factor, FliA, which is encoded by the Class II gene, *fliA* [13,14]. Expression of *fliA* is, in turn, governed by the master motility regulators, FlhD and FlhC. The FlhDC complex is encoded by Class I genes and is required for expression of all other flagellar genes [15-17]. Therefore, to determine the effect of CsrA on the upstream regulators of *yplA* and *fleB*, expression of *fliA* and *flhDC* was examined (Figure 3). Expression of *fliA* and *flhDC* were significantly less in the *csrA* mutant relative to wild-type. When *csrA* was reintroduced on a low copy plasmid into the *csrA* mutant, β-galactosidase activity was restored at all levels of the flagellar regulatory cascade. These results indicate CsrA affects *yplA* expression by activating the upstream regulatory genes *flhDC*. Furthermore, these results reveal CsrA affects motility by acting at the top of the regulatory hierarchy that affects flagellar gene expression.

The effect of CsrA on motility has been investigated in numerous bacterial species within the family of *Enterobacteriaceae*, which includes the *Yersiniae*. These studies indicate that regulation of motility by CsrA is not conserved. Mutation of *csrA* in *E. coli*, *Yersinia pseudotuberculosis* and *S.* Typhimurium results in loss of flagella [18-21]. In *E. coli*, it was demonstrated that regulatory control by CsrA occurs by binding to and stabilizing the transcript of the master regulator, *flhDC* [19,22]. This appears to also be true in *S.* Typhimurium since mutation of *csrA* results in decreased levels of *flhDC* mRNA [21]. Yet *S.* Typhimurium differs because CsrA appears to additionally modulate motility by affecting expression of *hilD*, a master regulator of virulence genes including those required for motility, and STM1344, a negative regulator of motility [21,23]. CsrA in *Y. pseudotuberculosis* also directly regulates motility by binding to *flhDC* transcript [20]; yet control of motility by the Csr system in this bacterium also differs because CsrA additionally acts indirectly through activation of *rovM*,an activator of motility [20,24]. It is possible that the same levels of regulatory control occur in *Y. enterocolitica* since there is a *rovM* homologue present in the genome. In contrast, CsrA (RsmA) in *Erwinia carotovora* negatively regulates production of flagella by destabilizing the mRNA transcripts of *flhDC* and *fliA* [25]. Thus, even within the family *Enterobacteriaceae*, the role of CsrA in coordinating physiology is highly malleable. Further diversity in the effect of CsrA on motility is seen in *Helicobacter pylori*, where CsrA is also required for motility [26]; however, the defect is not due alterations in the amount of major flagellin proteins or assembled flagellar structures. Instead, CsrA appears to either act at a relatively late stage in the motility regulatory hierarchy or affect the ability to use flagella. The diversity seen in how CsrA affects motility exemplifies how the Csr regulon has been differentially shaped to fit the varied lifestyles of bacteria.

C) Mutation of *csrA* in *Y. enterocolitica* results in sensitivity to sodium chloride and other osmolytes.

**Figure 4:**
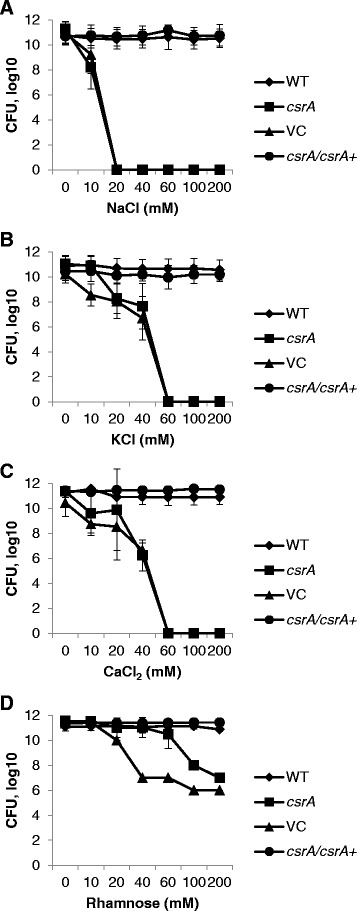
**Influence of sodium chloride and other osmolytes on colony formation of** ***Y. enterocolitica csrA*** **mutant.** Strains GY123 (WT), GY448 (*csrA*), GY6536 (VC) and GY6535 (*csrA/csrA+*) were cultured and serial dilutions plated onto TYE agar medium with indicated concentrations of **A)** NaCl, **B)** KCl, **C)** CaCl_2_ or **D)** rhamnose. Results represent average number of CFU ± standard deviation from at least three independent experiments performed in triplicate. Statistical analysis was performed using repeated measures two-way ANOVA.

Among the original observations that distinguished GY448 was that it grew on TYE medium but not on LB. The only difference between these two media is the inclusion of 90 mM sodium chloride in LB. To determine the concentration of sodium chloride that led to growth attenuation of the *csrA* mutant, wild-type and *csrA* mutant strains were cultivated on TYE agar with added sodium chloride at 0, 10, 20, 40, 60, 100 and 200 mM (Figure 4A). The *csrA* mutant was significantly inhibited in the ability to form colonies compared to wild-type when plated on media containing as little as 10 mM sodium chloride (p < 0.0001). Complementation of *csrA* completely restored growth at all concentrations of sodium chloride tested. The cloning vector had no effect on the phenotype of the *csrA* mutant. This growth attenuation was due to a bacteriostatic effect. Bacteria cultivated in TYE, collected and resuspended in a medium containing 300 mM sodium chloride were used to determine if the *csrA* mutant could be recovered on TYE agar. There was no significant difference in the ability of the *csrA* mutant and wild-type strains to recover and grow on TYE agar after 30 minutes, one hour, two hours, eight hours or 24 hours of sodium chloride exposure (data not shown). These results indicate that *Y. enterocolitica* with mutation in *csrA* is sensitive to even low concentrations sodium chloride and that this effect is bacteriostatic.

While the regulatory mechanisms of the Csr system have been well studied, the environmental signals that this complex system responds to remain somewhat obscure [6,8,9,27,28]. It is clear that quorum sensing and environmental pH are important [27,29,30]; however the influence of osmolarity has not been investigated. Therefore, we further probed whether the limitation of growth of the *csrA* mutant due to sodium chloride might be an effect caused by anionic identity, ionic strength or osmolarity. To distinguish the contribution of the anion, the effect of a monovalent salt, a divalent salt and a non-metabolizable carbohydrate were determined (Figure 4B, C and D). It was observed in each case that the *csrA* mutant displayed growth attenuation and the severity of the effect was titratable. Treatment with potassium chloride and calcium chloride indicated that significantly fewer *csrA* mutant colonies formed at 20 mM (p < 0.0001) and 40 mM (p < 0.0001), respectively (Figure 4B and C). At concentrations of 60 mM for both salts, the *csrA* mutant completely lost the ability to form colonies. Treatment with rhamnose did not eliminate colony formation of the *csrA* mutant at any concentration examined and significantly fewer colonies, relative to wild-type, formed at 100 mM (p < 0.0001) (Figure 4D). Complementation of *csrA* on a low-copy plasmid completely restored the ability of the *csrA* mutant to form colonies in all cases. These results suggest that high osmolarity is, at least, one element of stress that limits growth.

It is interesting to consider how sensitivity to high ionic strength and high osmolarity may affect the study of *csrA* mutant bacteria. Studies in *E. coli* K-12 are routinely performed on LB medium, using a strain with a transposon insertion located at codon 51, which reportedly retains partial CsrA activity [31,32]. Development of an *E. coli* K-12 *csrA* deletion mutant has been attempted but was unsuccessful [32]. This report showed that *csrA* was essential for growth on LB medium by demonstrating activation of an inducible plasmid encoding *csrA* could restore growth of a *csrA* deletion mutant on LB. It was noted that various growth conditions were used while attempting to make the mutant; however it is unclear whether variation in osmolarity was among them. Additionally, *S*. Typhimurium and *Y. pseudotuberculosis csrA* mutants are attenuated for growth on LB medium and *Pseudomonas aeruginosa rsmA* mutants are restricted for growth when cultured in nutrient yeast broth (NYB) or nutrient broth (NB). All of these media include 90 mM sodium chloride [20,23,33-35]. These reports of varying degrees of growth attenuation, in combination with the findings from this study, make it interesting to consider how CsrA affects bacterial responses to environmental osmolarity in different species. It is possible that CsrA affects both shared and species-specific signaling pathways that coordinate the bacterial response to osmotic cues. The effect of sodium chloride was not explored in the preceding reports; consequently, this study is the first to provide evidence that osmolarity of the growth medium may account for the previously observed phenotypes.

It is also noteworthy to consider how the osmolarity of media may impact experimental outcomes and analysis of the resulting data. For example, *Y. pseudotuberculosis* wild-type and *csrA* mutant strains were analyzed for expression of the virulence genes *rovA* and *rovM* when bacteria were cultured under two different growth conditions [20]. Bacteria were grown in either LB media that included the addition of 90 mM sodium chloride or in minimal media that contained 0.9 mM sodium chloride and 1 mM magnesium sulfate salts. Results of this study indicated that the Csr system affects expression of *rovA* and *rovM* in a media-dependent manner. While the media-dependent effect on expression of *csrA* itself was modest, the level of *csrC* expression was greatly reduced in minimal medium compared to LB medium. This suggests that medium-dependent regulation of virulence genes by the Csr system occurs through the control of CsrC levels which may change in response to salt concentration. The results of the current study in *Y. enterocolitica*, in combination with gene expression studies in *Y. pseudotuberculosis*, highlight how understanding the effect of the osmolarity of medium can be an important contributor to interpreting results obtained about the Csr system.

D) Mutation of *csrA* in *Y. enterocolitica* results in growth inhibition at 4°C and 42°C.

**Figure 5:**
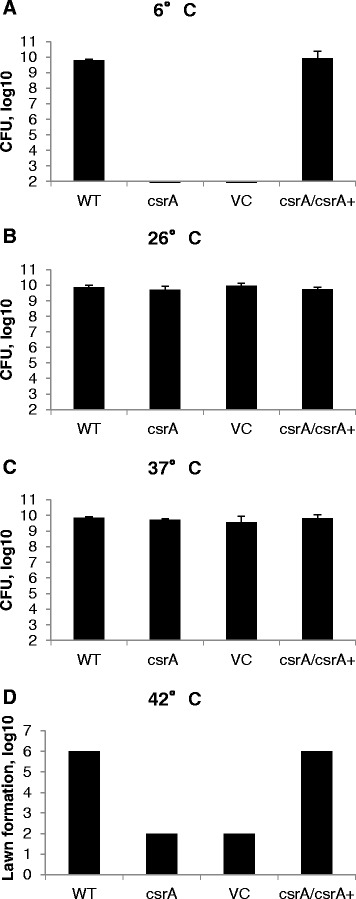
**The effect of temperature on growth of the** ***csrA*** **mutant of** ***Y. enterocolitica*** **.** Strains GY123 (WT), GY448 (*csrA*), GY6536 (VC) and GY6535 (*csrA/csrA+*) were cultured and serial dilutions were plated onto TYE agar medium in replicate. One replicate for each strain was incubated at **A)** 6°C for three weeks, **B)** 26°C for 48 hours, **C)** 37°C for 24 hours or **D)** 42°C for 24 hours. CFU **(A-C)** or lawn formation **(D)** was quantified. Results represent averages ± standard deviation from three independent experiments performed in triplicate.

Growth of many bacterial species is critical to control when it poses a threat to human health or contributes to any of a wide spectrum of economic losses [36]. Food-borne human pathogens and food spoilage microorganisms are most commonly constrained by storing food at low temperatures to minimize health risks, medical costs, food spoilage and recall of produce. Low temperatures affect bacterial growth by compromising membrane functions and reducing DNA replication, transcription and translation [36,37]. Yet some psychrotrophic bacteria are capable of growing despite low temperatures, which allows them to survive the cooling processes used within the food chain. For this reason, it is important to understand how bacteria respond to temperature as an environmental signal. *Yersiniae* are of particular concern within food systems because they can grow at a wide range of temperatures, from as low as 5°C to as high as 42°C [38]. To examine whether CsrA affects growth of *Y. enterocolitica* at different temperatures important for food safety, wild-type and *csrA* mutant bacteria were grown to stationary phase and plated on TYE. Bacteria were incubated at 6°C, 26°C, 37°C and 42°C and bacterial growth was quantified (Figure 5). When incubated at 26°C or 37°C, there was no significant difference between the number of colonies formed from cultures of wild-type and *csrA* mutant bacteria (Figure 5B and C). However, at 6°C the *csrA* mutant was unable to grow (Figure 5A). To determine whether the bacteria remained viable, these plates were subsequently transferred to the more favorable growth temperature of 37°C. This revealed a three log reduction in colony forming units (CFU) for the *csrA* mutant relative to wild-type (data not shown), indicating incubation at 6°C results in some lethality. The ability of bacteria to grow at 42°C was also examined. At this temperature, *Y. enterocolitica* does not form individual colonies. However, a threshold at which the collective population would form a lawn on the plate was observed. Using this as the criterion, there was a four log reduction in growth of the *csrA* mutant relative to wild-type (Figure 5D). These combined results reveal *csrA* is essential for growth of *Y. enterocolitica* at both the low and high ends of the temperature spectrum at which it can grow.

Within scientific literature, it is evident that the Csr system plays an important role in regulating responses of many bacterial species to temperature. A *csrA* mutant of *S.* Typhimurium was severely impaired for colony formation at 10°C, 15°C and 21°C, but not at 37°C [39]. Mutation of *csrA* in *H. pylori* had no effect on bacterial viability in response to heat shock; however, in a *csrA* mutant, transcription of genes involved in the heat shock response was altered [26]. Also, expression of *csr* genes themselves are differentially regulated over a broad range of temperatures in *E. coli* and *Legionella pneumophila* [40,41]. In combination with results of the current study, these reports suggest CsrA may be an important target for investigating temperature-dependent growth of bacterial species, including those that are of significant human health and economic importance.

E) Mutation of *csrA* in *Y. enterocolitica* results in increased sensitivity to antibiotics.

**Figure 6:**
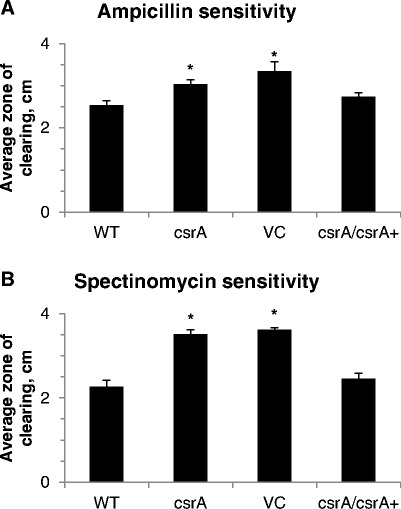
**Susceptibility of** ***Y. enterocolitica*** **strain JB580v and GY448 to ampicillin and spectinomycin.** Strains GY123 (WT), GY448 (*csrA*), GY6536 (VC) and GY6535 (*csrA/csrA+*) were cultured and bacteria were spread onto TYE agar medium. A disk containing **A)** 100 mg/ml ampicillin or **B)** 50 mg/ml spectinomycin was placed in the center of the plate. After incubation at 26°C for 48 hours, the diameter of the zone of growth inhibition around the disk was measured. Results represent averages ± standard deviation from three independent experiments performed in duplicate. *The p-value compared to WT was < 0.0001.

Considering the various of physiological changes that are modulated by CsrA, we speculated the *csrA* mutant may be altered in ways that affect susceptibility to antibiotics. Therefore, to investigate a broader range of functions that the loss of *csrA* may affect, we examined two different classes of antibiotics, a cell wall synthesis inhibitor, ampicillin, and a protein synthesis inhibitor, spectinomycin (Figure 6 and Table 1). The sensitivity of wild-type and *csrA* mutant bacteria to these antibiotics was investigated using a disk diffusion assay (Figure 6). The zone of growth inhibition around a disk containing 100 mg/ml ampicillin or 50 mg/ml spectinomycin was significantly larger for the *csrA* mutant relative to wild-type, indicating mutation of *csrA* increased susceptibility of *Y. enterocolitica* to these antibiotics. Another measure of sensitivity to antibiotics, minimum inhibitory concentration (MIC) testing, was also used (Table 1). The MIC of ampicillin was 16-fold greater for wild-type than the *csrA* mutant. Furthermore, consistent with results from the disk diffusion assay, the MIC of spectinomycin was at least fourfold greater for wild-type than the *csrA* mutant, since the *csrA* mutant was inhibited by the lowest concentration of spectinomycin examined. These experimental outcomes indicate mutation of *csrA* in *Y. enterocolitica* results in increased susceptibility to two antibiotics with different mechanisms of action. It is not apparent from studies of CsrA in other bacterial species whether increased sensitivity to antibiotics has been observed in *csrA* mutant bacteria. This is the first report, to our knowledge, to indicate a role for CsrA in modulating bacterial responses to antibiotics. It is particularly noteworthy that CsrA controls physiological responses to antibiotics with different mechanisms of action. This finding, in combination with other results from this study, indicates CsrA is involved in controlling multiple regulatory cascades that coordinate bacterial responses to a wide variety of environmental cues.

**Table 1:** **Minimum inhibitory concentration (mg/L) of ampicillin and spectinomycin for** ***Y. enterocolitica*** **strain JB580v and GY448**

**Strain**	**Ampicillin**	**Spectinomycin**
WT	400	25
*csrA*	25	<6.25
VC	25	<6.25
*csrA/csrA+*	200	25

Strains GY123 (WT), GY448 (*csrA*), GY6536 (VC) and GY6535 (*csrA/csrA+*) were grown to 0.5 McFarland standard in TYE and inoculated into TYE broth containing concentrations of ampicillin or spectinomycin diluted 1:2 and ranging from 400 mg/ml to 6.25 mg/ml. MIC was determined based on visible growth in samples after incubation for 36 hours at 26°C. Results from three independent experiments performed in duplicate.

## Conclusions

This study compiled evidence that CsrA has the ability to function differently in closely related species of bacteria and showed it modulates a diverse array of physiological processes in *Y. enterocolitica*. These findings may reflect bacterial lifestyle characteristics. *Y. enterocolitica* has an unusual ability to grow at low temperatures that occur during its free-living stages in terrestrial environments which are interrupted by episodes of parasitic growth as a pathogen in mammalian hosts. Also based on the results of this study, it is interesting to consider how alterations in temperature, osmolarity and antibiotic concentrations may be useful tools on a practical level. It is possible that modification of growth conditions may aid development of *csrA* mutant strains and help circumvent issues related to growth attenuation of *csrA* mutants.

## Methods

### Bacterial strains and media

The bacterial strains used in this study are described in Table 2. Strains of *Escherichia coli* were routinely grown at 37°C in Luria-Bertani (LB) broth (1% tryptone, 0.5% yeast extract, and 90 mM NaCl) or on LB agarose (Difco, Detroit, MI). *Yersinia enterocolitica* strains were grown at 26°C in Tryptone-Yeast Extract (TYE) broth (1% tryptone, 0.5% yeast extract) or on TYE agarose (Difco, Detroit, MI). Semisolid medium for the examination of motility contained 0.3% Difco agar. Phospholipase indicator agar (PLA) consisted of TYE medium supplemented with 1% Tween 80 and 1 mM CaCl_2_ [4] and solidified with 2% (wt/vol) agarose (Difco, Detroit, MI). Antibiotics (in micrograms per milliliter) were used as follows. For *Y. enterocolitica*, working concentrations were chloramphenicol, 10; kanamycin, 50; nalidixic acid, 20 and tetracycline, 7.5. For *E. coli*, working concentrations were chloramphenicol, 25; kanamycin, 50 and tetracycline, 15.

**Table 2:** **Bacterial strains and plasmids used in this study**

**Bacterial strain or plasmid**	**Genotype or relevant characteristics**	**Source or reference**
*Y. enterocolitica* strains		
JB580v	Serogroup O:8, Nal^r^ Δ*yenR* (R^−^, M^+^)	[42]
GY448	*csrA*::Tn*Mod*-RKm’	[3]
GY6536	*csrA*::Tn*Mod*-RKm’ pTM100	This study
GY6535	*csrA*::Tn*Mod*-RKm’ *csrA*::pTM100	This study
GY6575	*csrA*::Tn*Mod*-RKm’ *flhDC*::pFUSE	This study
GY6576	*csrA*::Tn*Mod*-RKm’ *fleB*::pFUSE	This study
GY6577	*csrA*::Tn*Mod*-RKm’ *fliA*::pFUSE	This study
GY6578	*csrA*::Tn*Mod*-RKm’ *yplA*::pFUSE	This study
GY6579	*csrA*::Tn*Mod*-RKm’ *csrA*::pTM100 *flhDC*::pFUSE	This study
GY6581	*csrA*::Tn*Mod*-RKm’ *csrA*::pTM100 *fleB*::pFUSE	This study
GY6582	*csrA*::Tn*Mod*-RKm’ *csrA*::pTM100 *fliA*::pFUSE	This study
GY6583	*csrA*::Tn*Mod*-RKm’ *csrA*::pTM100 *yplA*::pFUSE	This study
GY6584	*csrA*::Tn*Mod*-RKm’ pTM100 *flhDC*::pFUSE	This study
GY6585	*csrA*::Tn*Mod*-RKm’ pTM100 *fleB*::pFUSE	This study
GY6586	*csrA*::Tn*Mod*-RKm’ pTM100 *fliA*::pFUSE	This study
GY6587	*csrA*::Tn*Mod*-RKm’ pTM100 *yplA*::pFUSE	This study
*E. coli* strains		
DH5α	F^−^ φ80*lacZ*∆M15 ∆(*lacZYA*-*argF*)U169 *recA1 endA1 hsdR17*(r_k_^−^, m_k_^+^) *phoA supE44 thi-1 gyrA96 relA1* λ^−^	[43]
S17-1 λpir	*recA thi pro hsdR* ^−^ *hsdM* ^+^ RP4::2-Tc::Mu::Km Tn*7* λ*pir* (Tp^r^ Str^r^)	[44]
Plasmids		
pCR-Blunt II-TOPO	Km^r^	Invitrogen
pFUSE	Cm^r^, *mob* ^+^, *ori*R6K, suicide vector for transcriptional fusions to *lacZYA*	[45]
pTM100	mob^+^, derivative of pACYC184, Cm^r^ Tet^r^	[46]
pGY1298	pTM100 with a 0.5-kb fragment containing *csrA* not directional to *cat* promoter	This study
pGY714	pFUSE with *flhDC* promoter fragment	[47]
pGY716	pFUSE with *fleB* promoter fragment	[47]
pGY715	pFUSE with *fliA* promoter fragment	[47]
pGY713	pFUSE with *yplA* promoter fragment	[2]

### Characterization of transposon insertions sites and DNA sequencing

Chromosomal DNA was isolated from mutants that contained Tn*Mod*-RKm’ insertions and was digested with *Eco*RI. The digested DNA was ligated overnight, and replicating plasmids were recovered by electroporation of *E. coli* S17-1 λ*pir* followed by selection for kanamycin resistance [48]. Direct cloning of the transposon/chromosome junction was facilitated by the presence of a conditional *ori*R6K in Tn*Mod*-RKm’ that can function in specialized *E. coli* strains that carry a copy of *pir* [44]. Plasmids were isolated and analyzed by restriction digest to confirm the integrity of the transposon sequences. The sequence of the chromosomal DNA immediately adjacent to the transposon was then determined using primers that annealed near the ends of Tn*Mod*-RKm’ (primer KM1, 5′ – CCCCGAGCTCTTAATTAA – 3′, and primer KM2, 5′ – GAACACTTAACGGCTGAC – 3′). DNA sequence was obtained using an ABI Prism® 3730 Genetic Analyzer and BigDye® Terminator v. 3.1 Cycle Sequencing Kit with Gel Company Better Buffer (Applied Biosystems Inc., Foster City, CA).

### Construction of plasmids and bacterial strains

General DNA manipulations were done as described previously [49]. PCR-based DNA amplification of the chromosomal region containing *csrA* from wild-type *Y. enterocolitica* JB580v was performed using *PfuTurbo* DNA polymerase (Stratagene, La Jolla, CA). PCR used upstream primer *csrA*1 (5′ – CAATGCGCCATATCTCTATG – 3′) and downstream primer *csrA*3 (5′ – GTAACACGAGACGCTTCTTC – 3′), resulting in a 442 base-pair fragment of the region of the *Y. enterocolitica* chromosome from −232 to +210 relative to the translational start site of *csrA*. The DNA fragment was gel purified using a QIAquick gel extraction kit (Qiagen). The purified PCR product was initially cloned into pCR-Blunt II-TOPO (pTOPO) according to the manufacturer’s instructions (Invitrogen, Grand Island, NY). Plasmid DNA was isolated from *E. coli* DH5α using a QIAprep spin miniprep kit (Qiagen, Valencia, CA). The DNA fragment was then released by digestion with *Eco*RI (New England Biolabs, Ipswich, MA) for ligation into the same sites of the plasmid-based expression vector pTM100 [46] using T4 DNA ligase (New England Biolabs, Ipswich, MA). The nucleotide sequence of DNA generated by PCR was determined to confirm that there were no point mutations. DNA sequencing was performed at the DNA sequencing facility of University of California, Davis on an ABI Prism® 3730 Genetic Analyzer using BigDye® Terminator v. 3.1 Cycle Sequencing Kit with Gel Company Better Buffer (Applied Biosystems Inc., Foster City, CA). The direction of *csrA* to the promoter of the **c**hloramphenicol **a**cetyl**t**ransferase (*cat*) gene was investigated by PCR using vector primer *pTM1002HA*1 (5′ – GGATCCCTATCCCATATCACCAGCTC – 3′) and insert primer *csrA*1 (5′ – CAATGCGCCATATCTCTATG – 3′) or vector primer *pTM1002HA*3 (5′ – GTCGACCTATAACCAGACCGTTCAGC – 3′) and insert primer *csrA*3 (5′ – GTAACACGAGACGCTTCTTC – 3′). The resulting pTM100 plasmid with *csrA* oriented in the opposite direction of the *cat* gene, pGY1298, was introduced into *E. coli* S17-1λ(*pir*) by electroporation then mobilized into *Y. enterocolitica* strain GY448 by conjugation [50-52]. Previously constructed pFUSE plasmids were similarly mobilized from *E. coli* S17-1λ(*pir*) into the indicated strains of *Y. enterocolitica* by conjugation.

### Phospholipase assays

Phospholipase activity in a sample was determined by a modified radial-diffusion assay [53]. Individual colonies were streaked for isolation onto TYE plates containing 1% Tween 80 and 1 mM CaCl_2_ (PLA medium) [1,4]. Plates were imaged after incubation at 26°C for 48 hr. Phospholipase activity was detected as a zone of precipitation emanating from isolated colonies. Plates were imaged on a BioSpectrum Multispectral Imaging System (UVP, Upland, CA).

### Assays for motility

Phenotypic assays for motility were initiated by spotting a small portion of a colony at the center of fresh TYE plates containing 0.3% agar, as described previously [17]. After 48 hours incubation at 26°C, plates were imaged on a BioSpectrum Multispectral Imaging System (UVP, Upland, CA). Motility was scored as positive if the strains exhibited growth and migration emanating from the point of inoculation.

### Measurements of β-galactosidase activity

*Y. enterocolitica* harboring *lacZYA* ranscriptional fusions were cultured overnight at 26°C in TYE medium and subcultured to an OD_600_ of 0.1 in 5 ml TYE. Cultures were incubated at 26°C for 6 hours. Bacterial cells were harvested then assayed for β-galactosidase activity as previously described [54].

### Osmolyte and temperature sensitivity assays

*Y. enterocolitica* was grown in TYE medium at 26°C overnight. Serial dilutions were plated in triplicate onto plates containing TYE medium. For osmolyte assays, plates with added NaCl, KCl, CaCl_2_ or rhamnose at final concentrations of 10 mM, 20 mM, 40 mM, 60 mM, 100 mM and 200 mM were also used. Plates were incubated at 26°C for 48 hours except where noted for temperature sensitivity assays, in which case replicate plates were also incubated at 6°C for three weeks, 37°C for 24 hours and 42°C for 24 hours. Subsequently, colony forming units were quantified, with the exception of plates incubated at 42°C. At this temperature, bacteria were unable to form individual colonies at any dilution. Bacteria either exhibited no growth or growth in the form of a lawn. Therefore, bacterial growth was reported as the dilution at which a lawn was present (threshold lawn formation).

### Antibiotic susceptibility assays

*Y. enterocolitica* was grown in TYE medium at 26°C overnight. Sterile cotton swabs were used to distribute bacteria evenly across the surface of TYE agar medium. A disk containing either 100 mg/ml ampicillin or 50 mg/ml spectinomycin was placed in the center of the plate and incubated at 26°C for 48 hours. The diameter of the zone of clearing around the disk was measured in centimeters. Determination of minimum inhibitory concentration (MIC) of ampicillin and spectinomycin was also performed as previously described [55]. Briefly, indicated strains were grown to 0.5 McFarland standard and inoculated into TYE broth containing 1:2 dilutions of ampicillin or spectinomycin. MIC was determined based on visible growth after incubation for 36 hours at 26°C.

### Statistical analysis

Data were analyzed using one-way between subjects analysis of variance and Tukey’s post hoc test, except where indicated, to determine statistically significant p-values at p < 0.05.

## Abbreviations

CFU: Colony forming units
CsrA: Carbon storage regulator A
LB: Lauria-Bertani
*orf*: Open reading frame
MIC: Minimum inhibitory concentration
T3SS: Type three secretion system
TYE: Tryptone yeast extract
VC: Vector control plasmid pTM100
WT: Wild-type
Yex: YplA export phenotype
